# *GSTT1/GSTM1* Genotype and Anti-Tuberculosis Drug-Induced Hepatotoxicity in Peruvian Patients

**DOI:** 10.3390/ijms231911028

**Published:** 2022-09-20

**Authors:** Luis Jaramillo-Valverde, Kelly S. Levano, David D. Tarazona, Andres Vasquez-Dominguez, Anel Toledo-Nauto, Silvia Capristano, Cesar Sanchez, Eduardo Tarazona-Santos, Cesar Ugarte-Gil, Heinner Guio

**Affiliations:** 1 Laboratorio de Biotecnología y Biología Molecular, Instituto Nacional de Salud, Lima 15046, Peru; 2 School of Medicine, Universidad Continental, Lima 15046, Peru; 3 INBIOMEDIC Research and Technological Center, Lima 15046, Peru; 4 Departamento de Genética, Ecologia e Evolução, Instituto de Ciências Biológicas, Universidade Federal de Minas Gerais, Belo Horizonte 31270-901, MG, Brazil; 5 Instituto de Medicina Tropical Alexander von Humboldt, Universidad Peruana Cayetano Heredia, Lima 15102, Peru; 6 School of Medicine, Universidad Peruana Cayetano Heredia, Lima 15102, Peru; 7 School of Medicine, Universidad Cientifica del Sur, Lima 15067, Peru

**Keywords:** tuberculosis, GSTT1, GSTM1, hepatotoxicity

## Abstract

In Peru, 24,581 people were diagnosed with tuberculosis (TB) in 2020. Although TB treatments are effective, 3.4–13% are associated with significant adverse drug reactions (ADRs), with drug-induced liver injury (DILI) considered the most predominant. Among the first-line antituberculosis drugs, isoniazid (INH) is the main drug responsible for the appearance of DILI. In the liver, INH is metabolized by the enzymes N-acetyltransferase-2 (NAT2), cytochrome P450 2E1 (CYP2E1), and glutathione S-transferase (GST) with two isoforms, GSTT1 and GSTM1. Based on previous studies, we hypothesized that interactions between the *GSTT1* and *GSTM1* null genotypes induce DILI in TB patients. In this cross-sectional study of 377 participants who completed their anti-TB treatment, we genotyped by revealing the presence or absence of 215- and 480-bp bands of *GSTM1* and *GSTT1*, respectively. We found that the prevalence of the *GSTM1* genotype was 52.79% and 47.21% for presence and null, respectively, and for *GSTT1* it was 69.76% and 30.24% for presence and null, respectively. Neither genotype was prevalent in the patients who developed DILI (*n* = 16). We did not confirm our hypothesis; however, we found that the combination of *GSTM1* present genotype, *GSTT1* null genotype, fast *NAT2* acetylators, and *CYP2E1* c1/c1 genotype had a significant risk for the development of ADR (OR 11; *p* = 0.017; 95% CI: (0.54–186.35)). We propose that the presence of the *GSTM1* present genotype, *GSTT1* null genotype, fast *NAT2* acetylators, and *CYP2E1* c1/c1 genotype in the Peruvian population could be considered a risk factor for the development of ADR due to therapeutic drug intake.

## 1. Introduction

At the liver level, isoniazid (INH) is acetylated by N-acetyltransferase-2 (NAT2) to acetylhydrazine, and then oxidized to toxic intermediates by the enzyme cytochrome P450 2E1 (CYP2E1) [1]. The enzymes NAT2 and glutathione S-transferase (GST) participate in the detoxification of these toxic compounds produced through acetylation and conjugation mechanisms, respectively. Some risk factors for adverse drug reactions (ADRs) have already been reported, such as coinfection with human immunodeficiency virus (HIV), hepatitis B and C, advanced age, and female gender [2,3]; however, they vary according to the genetic population characteristics of the people.

For this reason, it is necessary to study genetic factors and their consequences in treatment. In antituberculosis treatment, acetylation by NAT2, followed by oxidation by CYP2E1, is considered a candidate process for the generation of hydrazine, which is a reactive metabolite [4]. Some studies suggest that genetic polymorphisms in NAT2 and CYP2E1 are associated with ADRs [5]. However, other studies do not reach this conclusion [6,7].

The detoxification of INH metabolites is very important, and the enzymes GSTM1 and GSTT1 are essential in this process; this is because the enzyme GST catalyzes the conjugation of species with glutathione [8]. Glutathione undergoes nucleophilic attack and is catalyzed by GST, converting it to electrophilic substrates, reducing the reactivity in cellular macromolecules with potential toxic compounds [9]. Two highly polymorphic GST isoforms have been reported: on chromosome 1p13.3 the GTM1 gene encodes the GSTµ isoform and on chromosome 22q11.2 the *GSTT1* gene encodes the GSTθ isoform [10]. It has been reported that the enzymatic activity loses its function when homozygous deletions are present in *GSTM1* and *GSTT1*, causing the loss of glutathione [11].

The *GSTM1* and *GSTT1* null genotypes have been associated with the presence of ADRs in some studies; however, there is still no consensus on this. Previous studies revealed an association between the presence of ADRs and the null or deleted genotype of *GSTM1* [12,13]; however, this association has been ruled out by more recent studies [14,15]. Similarly, few studies have reported an association between *GSTT1* null genotypes and ADRs [15], while a few others find no statistically significant association [13,14,16]. Therefore, the role of the *GST* polymorphism in the development of ADRs remains unclear among diverse populations, and even more so among ethnic groups.

Individual and population behavior is influenced by genetic–environmental factors; therefore, in order to understand human genetic variations, it is important to study human genetic diversity through its geographic distribution [17]. That is the value of studying the American population with a pharmacogenomic approach due to the interethnic mixture [18]. For this reason, pharmacogenomics is aimed at understanding how pharmacological therapy is affected by our genetic diversity, identifying the genotype of an individual to predict their metabolism against different drugs, maximizing the probability of treatment success, and minimizing the presence of adverse reactions; this is framed as personalized medicine [19,20,21]. Although metabolic genotypes and phenotypes have been extensively studied in Caucasian and Asian populations, this information is still lacking in most Latin American populations. Only three investigations related to pharmacogenomics have analyzed native Peruvian samples [22,23,24]. With the high prevalence of tuberculosis (TB) in Peru, research is focusing on demonstrating the role of pharmacogenomics in the treatment of TB, starting with the determination of the genetic polymorphisms that affect the drugs INH and RIF and, in this way, affect efficient treatment and collaborate with the appearance of adverse effects. In the previous study, we reported *NAT2* and *CYP2E1* data in other populations of the American continent and the rest of the world [24].

The objective of this study is to determine the prevalence of, and evaluate the association between, the genetic polymorphisms of *GSTM1* and *GSTT1* and the presence of ADRs. The identification of genetic markers to predict the susceptibility to develop ADRs should serve to improve the management and control of TB. The current study is also important in terms of the great challenges in treating the high incidence of tuberculosis in our country. Peru is made up of diverse ethnic origins which lead to the presence of genetic heterogeneity [25]. To the best of our knowledge, this is the first study to identify *GST* gene polymorphisms as plausible risk factors in the development of anti-TB drug toxicity in the Peruvian population.

## 2. Results

No patient was excluded from the study. This cohort was predominantly male (55%). A total of 16 out of 377 participants (4.1%) were diagnosed with DILI, a mild ADR type with symptoms that included nausea and vomiting/gastric pain. Table 1 shows the frequency distribution of both biological and clinical variables. 

The prevalence of *GSTM1* genotypes in the study population was 52.79% and 47.21% for presence and null, respectively. In the case of *GSTT1*, the prevalence of genotypes was 69.76% and 30.24% for presence and null, respectively.

On the other hand, the *GSTM1* and *GSTT1* genotypes were not associated with DILI, and the allelic frequencies of both groups (with and without adverse reaction) were similar and were in Hardy–Weinberg equilibrium (*p* > 0.05), which suggests that these samples belonged to a population in genetic equilibrium. The chi-square test or, when necessary, Fisher’s exact test was used.

In Table 2, there is no association between the frequencies of genotypes and DILI. There is no evidence that the presence of variant genotypes of *GSTM1*, *GSTT1*, or their interaction, can become isolated risk or protective factors for developing ADRs during TB treatment (*p* > 0.05).

When the effects of combining *GSTM1, GSTT1,* and *CYP2E1* genotypes and NAT2 acetylator status were examined, it was found that patients who were genotype present *GSTM1*, genotype null *GSTT1*, fast NAT2 acetylators, and had genotype *CYP2E1* c1/c1 had a significant risk (OR = 11; *p* = 0.017) for the development of DILI compared to the most prevalent combination between *GSTM1, GSTT1, NAT2* and *CYP2E1* genotypes (Table 3).

## 3. Discussion

GST is an important component of phase II drug-metabolizing enzymes involved in the removal of toxic metabolites. There are several isoforms of the GST gene; the *GSTM1* and *GSTT1* genotypes are highly polymorphic among various ethnic groups, as well as within relatively homogeneous ethnic groups [26,27]. 

Our study in the Peruvian population analyzed the association between *GSTM1/GSTT1* genotypes and hepatotoxicity induced by anti-TB drugs. Absence of GSTM1 and GSTT1 activity caused by homozygous null mutations or deletions has been implicated in liver injury due to lack of protection against oxidative species [28]. The frequencies of *GSTM1* and *GSTT1* homozygous null genotypes in this study were in agreement with other studies in the Brazilian population [6], demonstrating the consistency of our data. 

In the total population, the *GSTT1* present genotype was the most prevalent (69.76%), and there was no significant difference between patients with and without adverse reaction to TB, which is consistent with other reported studies [14,28]; however, a study conducted in Caucasians found a significant association between *GSTT1* null genotypes and antituberculosis drug-induced hepatotoxicity (*p* = 0.03) [15].

The number of patients with null *GSTM1* genotypes with adverse reactions was higher than that of patients with present genotype *GSTM1* with adverse reaction; although we did not observe a significant association between null genotypes of *GSTM1* and anti-TB DILI, as reported in a few other studies [14,29]. As we saw earlier, *GSTM1* genotypes are highly polymorphic or variable between different ethnic groups, even within relatively homogeneous ethnic groups [26].

The combined deletion of the *GSTM1* and *GSTT1* gene has been reported in previous studies [30,31]. In the present study, the percentage of individuals with both *GSTM1* and *GSTT1* null mutations was higher in DILI total patients (6%); however, no statistically significant difference was found, in agreement with previous studies that did not find a significant association for *GSTM1* null and *GSTT1* null in Indian (*p* = 0.39) and Caucasian (*p* = 0.17) patients with the presence of anti-TB DILI [14,15].

This study is the first to our knowledge that examines the effects of combining *GSTM1*, *GSTT1*, and *CYP2E1* genotypes, NAT2 acetylator status, and the presence of adverse reactions in the Peruvian population. This study found an interesting result: the present *GSTM1* genotype, *GSTT1* null genotype, fast NAT2 acetylators, and *CYP2E1* c1/c1 genotype had a significant risk for the development of ADR and is suspected to be a risk factor for INH-induced hepatotoxicity.

Few studies have observed the relationship between GST genotypes and hepatotoxicity induced by antituberculosis drugs [12,15,32]. For example, Roy et al. (2001) have observed a significant association between the *GSTM1* homozygous null genotype and anti-tuberculous drug-induced hepatotoxicity in Indian tuberculosis patients. Huang et al. (2007) have found similar results in a Chinese population. However, the presence of the *GSTT1* homozygous null genotype was similar between cases and controls in both studies.

On the other hand, the same analysis in Spanish patients with TB showed an opposite effect; the homozygous null *GSTT1* genotype was highly associated with anti-TB drug-induced hepatotoxicity, and no significant associations were found between the homozygous null *GSTM1* genotype and hepatotoxicity [15]. This difference can be explained because concentrations of antituberculosis drugs and genetic polymorphisms vary significantly between populations and/or individuals [33].

In addition, the information obtained in the present study contributes to affirm that the allelic frequency of *GSTM1* and *GSTT1* is different according to the geographic location of the populations [34]. This reinforces the importance of pharmacogenomic studies in an ethnically diverse population such as Peru. Due to the controversy presented by the association between *GSTM1*–*GSTT1* polymorphisms and ADRs, three meta-analysis studies revealed that there is a significant risk of ADRs attributed only to the *GSTM1* null genotype [28,35,36].

This discrepancy could be due to differences in the study designs of the compared projects, as well as in the ethnic origin of the populations studied; additionally, it could be attributed to several factors, such as different metabolism rate and disposition, ability to detoxify xenobiotics, susceptibility to certain diseases, socioeconomic status, and different acquired lifestyle. Furthermore, more studies with a larger population size are needed to confirm our findings [5].

This study also had the limitations of an observational study with tuberculosis patients attending a routine healthcare setting. Drug-related exposures recorded by the treating physician were analyzed. The incidence of hepatotoxicity induced by antituberculosis drugs is very low; however, our study was multicentered to better represent the population studied and to include as many cases of DILI as possible. Therefore, a prospective cohort study in TB patients with or without ADRs could confirm our findings.

## 4. Methods and Materials

***Studied population and participants*:** Our study included 377 unrelated individuals, a subgroup of patients diagnosed with pulmonary tuberculosis between 2014 and 2015, recruited from health establishments of the Minister of Health (MINSA) located in Lima and Callao-Perú using previously collected data [24].

The inclusion criteria were patients: (i) with daily treatments of isoniazid, rifampicin, pyrazinamide and ethambutol for 2 months, followed by 4 months treatment of isoniazid and rifampicin, with drug dosages calculated according to bodyweight; (ii) with normal serum alanine aminotransferase (ALT), aspartate aminotransferase (AST), and bilirubin levels, no symptoms related to abnormal liver function (i.e., jaundice) prior to anti-TB drug treatment, and close monitoring of changes in liver function within 2 months of treatment; and (iii) with and without hepatotoxicity during drug treatment.

Patients with any of the following conditions were excluded from the study: (i) malnutrition; (ii) human immunodeficiency virus type 1 (HIV) infection; (iii) alcoholic liver disease or habitual drinking; (iv) hepatitis B or C infection, liver disease, systemic diseases and/or treatment with drugs other than the anti-TB drugs that can induce hepatotoxicity. All 377 participants (207 males and 170 females) completed their anti-TB treatment.

***Patient Consent Statement:*** This study was approved by the Ethics in Research Committee of the Peruvian National Institute of Health (OI-087-13) and by Universidad Peruana Cayetano Heredia (SIDISI: 201091). Informed consent was obtained from all the participants. 

***Study design and data collection*:** This was a cross-sectional and observational study, with 377 tuberculosis patients, of both genders, aged between 15 and 50 years, which was the age group with the highest tuberculosis prevalence and the economically active one (possibility of infection by transport from one place to another) who lived in the study area [37]. 

We selected a subpopulation with the most prevalent sociodemographic characteristics of the general population. Our study was multicentered to better represent the population studied and to enroll as many DILI cases as possible.

*Data collection sheet:* This was designed to make it possible to collect sociodemographic characteristics, as well as clinical results, from medical records, in the studied population. The data collection sheet was accepted by the CIEI-INS. Two nurses, trained by the principal investigator, collected the information. The collected clinical records were analyzed by an epidemiologist and a biostatistician. 

*Blood Samples:* After the survey questionnaire, peripheral blood samples (4 mL) were obtained from all 377 studied patients at baseline.

**Figure 1:**
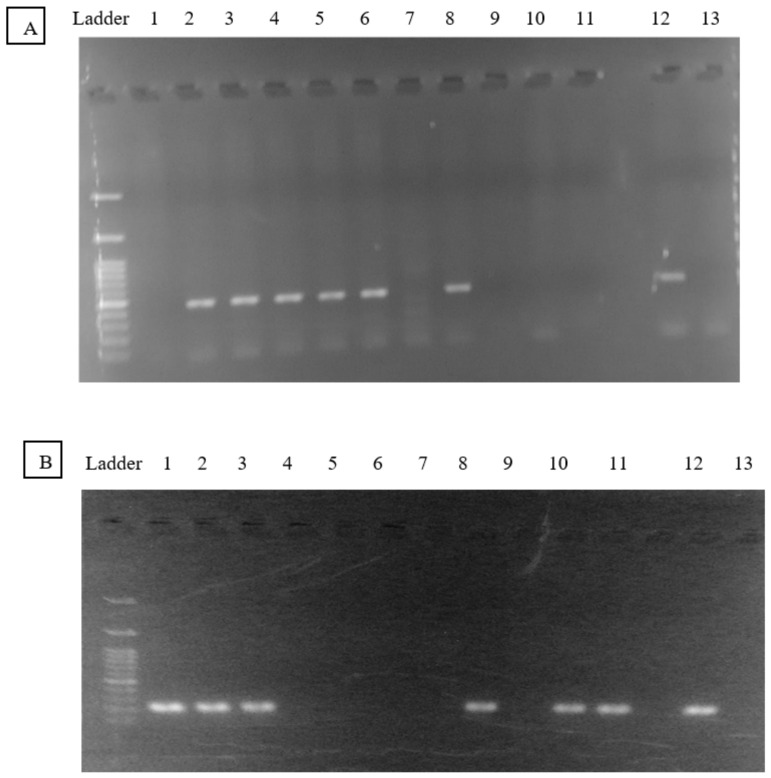
PCR products revealed using 1.5% agarose gel electrophoresis. The bands were 215 and 480 bp in size, depending on the GST genotype, designated as GSTT1 (**A**) and GSTM1 (**B**). Samples are 1–11, 12 positive amplification sample and 13 negative amplification sample.

***Laboratory methods:*** Genomic DNA was extracted from peripheral blood of all 377 participants using the genomic DNA extraction kit QIAamp DNA Blood Mini Kit (Qiagen, Germany) according to the manufacturer’s instructions. The selected genomic DNA regions for the analysis of each gene included *GSTT1* and *GSTM1* genes in 2 replicates. These regions were amplified by PCR using the Platinum Taq DNA polymerase kit (Invitrogen, USA) as described previously [15] with the following primers: 5′-GAACTCCCTGAAAAGCTAAAGC-3′ and 5′-GTTGGGCTCAAATATACGGTGG-3′ for the *GSTM1* gene and the specific primers 5′-TTCCTTACTGGTCCTCACATCTC-3′ and 5′-TCACCGGATCATGGCCAGCA-3′ for the *GSTT1* gene [15]. PCR was performed in a final volume of 25 mL, as follows: a 5-min denaturation at 94 °C, 30 cycles of 1 min at 94 °C, 1 min at 55 °C, and a final 5-min extension at 72 °C. The PCR products were revealed by the presence of bands using 1.5% agarose gel electrophoresis (gel prepared in 1X TAE buffer and stained with SYBR ^®^ Safe DNA). The bands were 215 and 480 bp in size, depending on the *GST* genotype, designated as *GSTM1* and *GSTT1* marker bands, respectively. All electrophoresis reports included known positive and negative samples for both genes (Figure 1).

***Measures and analysis****:* Presence of DILI (yes/no) was the outcome. This information was collected from the medical record of each patient and was diagnosed by a medical doctor. Liver profile is measured 2 months after starting treatment in all patients with sensitive tuberculosis in Peru [37]. Hepatotoxicity was defined as elevated aminotransferase levels and identified as being three times higher than before initiating TB treatment, with associated symptoms of hepatitis. Symptoms were considered as the occurrence of jaundice, nausea, vomiting, dyspepsia, and asthenia [38]. The reference values adopted were AST—36 UI/mL and ALT—32 UI/mL [39]. 

The *GST* genotype, exposure of interest, was classified as “wild and null”. The *GST* genotype is defined as the genetic information of the DNA fragments of the *GSTT1* and *GSTM1* genes to reduce the activity of potentially toxic compounds with cellular macromolecules. The other covariates were grouped into (1) demographics (gender and age), (2) alcohol consumption, (3) cholesterol, (4) hemoglobin, (5) glucose, and (6) body mass index (BMI). All the covariables were obtained from biological samples or at the time of interview of each patient.

NAT2 acetylator genotypes were classified as “slow, intermediate, and rapid,” and CYP2E1 genotypes were classified as “c1/c1, c1/c2, and c2/c2” in a previous study [29]. In this study, we found that rapid, intermediate, and slow NAT2 acetylators were 15%, 38%, and 47%, respectively, in the general population. Intermediate NAT2 acetylator is the least prevalent among patients with adverse reactions (*p* = 0.024). Finally, we found that the combination of intermediate NAT2 acetylators and CYP2E1 c1/c1 genotypes significantly protected (OR = 0.16; *p* = 0.049) against the development of DILI in our population [40].

*Statistical analysis.* A power of 92.6% was obtained when comparing the difference in the mean probability of the outcome variable between two categories of the independent variable (wild and null genotype). A ratio of 0.03 was assumed between wild and null genotypes and a probability of presence of RAFAs with wild and null genotypes, of 0.15 and 0.55, respectively, according to published data [5]. 

The chi–square test was used, or when necessary, Fisher’s exact test, to check the statistical significance of differences in the frequency distribution of variables and the Hardy–Weinberg equilibrium. Variables of interest had a normal distribution, so we calculated the median (interquartile), and used the Mann–Whitney test when it was necessary. Bivariate logistic regression analysis was performed, and the magnitude of the associations was expressed by the odds ratio (OR) as an estimate of relative risk, with a confidence interval of 95% regression. This strategy is correct when the prevalence of the disease or condition of interest being studied is small (ADRs: 3.4–13%), since the OR would give a value close to the prevalence ratio (PR) [41]. Data analysis was performed using Stata v15 (StataCorp, College Station, TX, USA) considering a statistical significance of *p* < 0.05.

## 5. Conclusions

We do not report evidence that the presence of variant genotypes of *GSTM1, GSTT1*, or their interaction, can become isolated risk or protective factors for developing ADRs during TB treatment. Although the present study is the first to examine the effects of combining *GSTM1, GSTT1,* and *CYP2E1* genotypes, NAT2 acetylator status, and the presence of adverse reactions, finding an interesting result, the present *GSTM1* genotype, *GSTT1* null genotype, fast NAT2 acetylators, and *CYP2E1* c1/c1 genotype had a significant risk for the development of ADR. 

This is the first pharmacogenetic study carried out in a mestizo population where the ancestral component is greater than 50% native. The findings based on the type of phenotype (slow or fast metabolizer) in relation to its genetic polymorphism, show that the results are different from those found in other populations with a different ancestral component.

## Figures and Tables

**Table 1:** Clinical and biological variables of adult patients diagnosed with sensitive pulmonary tuberculosis in Lima during the years 2014–2015.

	Total		Adverse Reaction (DILI)				
Variables			Yes		No		*p*-Value
	N	%	N	%	N	%	
**Sex**							
Male	207	54.91	10	62.50	197	54.57	
Female	170	45.09	6	37.50	164	45.43	0.533 ^a^
**Age (years)**	-	-	24.3 *	(20.3–30.8) †	24.2 *	(20.9–29.6) †	0.995 ^b^
**Alcohol consumption**							
No	49	13.00	3	18.75	46	12.74	
Yes	328	87.00	13	81.25	315	87.26	0.346 ^c^
**Cholesterol (mg/dL)**	-	-	154.0 *	(141.2–185.0) †	169 *	(149.0–189.0) †	0.214 ^b^
**Hemoglobin (g/dL)**	-	-	13.9 *	(12.2–15.6) †	13.6 *	(12.4–14.9) †	0.769 ^b^
**Glucose (mg/dL)**	-	-	89.5 *	(80.0–95.0) †	83.1 *	(77.9–91.1) †	0.084 ^b^
**BMI (kg/m^2^)**	-	-	21.1 *	(19.7–22.5) †	22 *	(20.3–23.6) †	0.182 ^b^
***GSTM1* genotype**							
Present	199	52.79	6	3.02	193	96.98	
Null	178	47.21	10	5.62	168	94.38	0.160 ^c^
***GSTT1* genotype**							
Present	263	69.76	10	3.8	253	96.20	
Null	114	30.24	6	5.26	108	94.74	0.346 ^c^

Variables in black; * Median; † (Q1–Q3); BMI (Body mass index); GST (glutathione S-transferase); Statistically significant (*p* < 0.05); ^a^ chi-square test; ^b^ Mann–Whitney test; ^c^ Fisher’s exact test.

**Table 2:** Association of the null genotype *GSTM1* and *T1* with the risk of DILI in the Peruvian population.

	Adverse Reaction (DILI)			
	Yes = 16 (%)	No = 361 (%)	OR (CI 95%)	*p*-Value
***GSTM1***				
Present (M+)	6 (3.02)	193 (96.98)	-	
Null (M−)	10 (5.62)	168 (94.38)	1.915 (0.61–6.54)	0.211
***GSTT1***				
Present (T+)	10 (3.80)	253 (96.20)	-	
Null (T−)	6 (5.26)	108 (94.74)	1.406 (0.41–4.39)	0.518
**Both *GSTM1* and *T1***			-	
M+/T+	13 (3.96)	314 (96.04)		
M+/T−	7 (5.47)	121 (94.53)	1.397 (0.46–3.87)	0.485
M−/T+	3 (4.92)	61 (95.08)	1.188 (0.21–4.50)	0.793
M−/T−	3 (6.00)	47 (96.00)	1.542 (0.27–5.90)	0.508

Variables in black; Data is represented as n (%); Statistically significant (*p* < 0.05); OR: odds ratio; CI: confidence intervals.

**Table 3:** Combined effects of NAT2, CYP2E1, and GST with the risk of DILI in the Peruvian population.

				Adverse Reaction (DILI)			
*GSTM1*	*GSTT1*	*CYP2E1*	NAT2	Yes = 16 (%)	No = 361 (%)	OR (IC 95%)	*p*-Value
Present	Present	C1/C1	Slow	3	33	Reference	
Null	Present	C1/C1	Slow	3	33	1 (0.12–8.02)	1
Present	Null	C1/C1	Slow	0	20	0 (0–2.28)	0.185
Null	Null	C1/C1	Slow	1	15	0.73 (0.01–10.09)	0.795
Present	Present	C1/C2 or C2/C2	Slow	0	27	0 (0–1.67)	0.124
Null	Present	C1/C2 or C2/C2	Slow	0	19	0 (0–2.4)	0.196
Present	Null	C1/C2 or C2/C2	Slow	0	14	0 (0–3.31)	0.265
Null	Null	C1/C2 or C2/C2	Slow	2	5	4.4 (0.28–47.68)	0.126
Present	Present	C1/C1	Intermediate	0	29	0 (0–1.55)	0.111
Null	Present	C1/C1	Intermediate	1	34	0.32 (0.01–4.33)	0.317
Present	Null	C1/C1	Intermediate	0	16	0 (0–2.86)	0.234
Null	Null	C1/C1	Intermediate	0	13	0 (0–3.58)	0.283
Present	Present	C1/C2 or C2/C2	Intermediate	0	15	0 (0–3.08)	0.249
Null	Present	C1/C2 or C2/C2	Intermediate	0	21	0 (0–2.16)	0.174
Present	Null	C1/C2 or C2/C2	Intermediate	0	7	0 (0–7.04)	0.428
Null	Null	C1/C2 or C2/C2	Intermediate	0	6	0 (0–8.36)	0.463
Present	Present	C1/C1	Rapid	0	14	0 (0–3.31)	0.265
Null	Present	C1/C1	Rapid	2	10	2.2 (0.16–21.78)	0.413
Present	Null	C1/C1	Rapid	2	2	11 (0.54–186.35)	0.017
Null	Null	C1/C1	Rapid	0	6	0 (0–8.36)	0.463
Present	Present	C1/C2 or C2/C2	Rapid	0	10	0 (0–4.75)	0.345
Null	Present	C1/C2 or C2/C2	Rapid	1	3	3.67 (0.05–65.57)	0.292
Present	Null	C1/C2 or C2/C2	Rapid	1	2	5.5 (0.07–129.91)	0.17
Null	Null	C1/C2 or C2/C2	Rapid	0	2	0 (0–28.51)	0.671

Data are represented as n (%); Statistically significant (*p* < 0.05); OR: odds ratio; CI: confidence intervals.
